# Disease activity–guided dose optimization including discontinuation of TNF inhibitors in rheumatoid arthritis is effective for up to 10 years: an observational follow-up of the DRESS study

**DOI:** 10.1093/rheumatology/keae103

**Published:** 2024-02-12

**Authors:** Céleste J T van der Togt, Nathan den Broeder, Marleen S Boonstra, Aatke van der Maas, Alfons A den Broeder, Noortje van Herwaarden

**Affiliations:** Department of Rheumatology, Radboud Institute for Health Sciences, Radboudumc, Nijmegen, The Netherlands; Department of Rheumatology, Sint Maartenskliniek, Ubbergen, The Netherlands; Department of Rheumatology, Radboud Institute for Health Sciences, Radboudumc, Nijmegen, The Netherlands; Department of Rheumatology, Sint Maartenskliniek, Ubbergen, The Netherlands; Department of Rheumatology, Sint Maartenskliniek, Ubbergen, The Netherlands; Department of Rheumatology, Sint Maartenskliniek, Ubbergen, The Netherlands; Department of Rheumatology, Sint Maartenskliniek, Ubbergen, The Netherlands; Department of Rheumatology, Sint Maartenskliniek, Ubbergen, The Netherlands; Division of Pharmacology and Toxicology, Department of Pharmacy, Radboud University Medical Centre, Nijmegen, The Netherlands

**Keywords:** rheumatoid arthritis, TNF inhibitors, adalimumab, etanercept, biologics, dose reduction, tapering

## Abstract

**Objective:**

The objective of this study was to investigate the safety and effectiveness of disease activity–guided dose optimization of TNF inhibitors in RA over 10 years.

**Methods:**

The study involved an observational long-term extension of a randomized study of participants who completed the 3-year extension of the DRESS-study. After the randomized phase (months 0–18), disease activity–guided dose optimization was allowed for all. The main outcomes were mean time-weighted DAS28-CRP; biologic and targeted synthetic DMARD (b/tsDMARD) use per year, as proportion of daily defined dose; proportion of patients reaching discontinuation; durability and effectiveness of subsequent dose reduction attempts; and radiographic progression between years 3 and 10 using the Sharp–van der Heijde score.

**Results:**

A total of 170 patients were included, of whom 127 completed the 10-year follow-up. The mean disease activity remained low (DAS28-CRP 2.13, 95% CI 2.10–2.16), while the b/tsDMARD dose reduced from 97% at baseline (95% CI 96–99%, *n* = 170) to 56% at year 10 (95% CI 49–63%, *n* = 127). Of 161 participants with an optimization attempt, 119 (74%) reached discontinuation with a median duration of 7 months (interquartile range 3–33 months), and 25 participants never had to restart their b/tsDMARD (21%, 95% CI 14–29%). The mean dose reduction after dose optimization was 48% (*n* = 159) for the first optimization attempt, and 33% for a subsequent attempt (*n* = 86). Of the 86 participants, 41 (48%) had radiographic progression exceeding the smallest detectable change (5.7 units), and progression was associated with disease activity, not b/tsDMARD use.

**Conclusion:**

Long-term disease activity–guided dose optimization of TNF inhibitors in RA, including discontinuation and multiple tapering attempts, remains safe and effective.

Rheumatology key messages

- Disease activity–guided dose optimization of TNF inhibitors is safe and effective for up to 10 years, resulting in low stable disease activity while using approximately half of the b/tsDMARD dose.
- A discontinuation attempt is supported by this study, which also found that subsequent dose optimization attempts after ∼2.5 years could lead to further dose reduction.
- As radiological progression is associated with mean disease activity, a high-quality treat-to-target approach is important when tapering.

## Introduction

TNF inhibitors (TNFis) have shown to be effective and safe in the treatment of RA [1], but have drawbacks, including infusion or injection site reactions, a somewhat higher risk of serious infections [2], and high costs [3]. In patients with controlled RA, these drawbacks can be reduced using disease activity–guided dose optimization. This includes a stepwise decrease of the TNFi dose (with or without discontinuation as a final step) together with treat-to-target, so that treatment can be intensified in case of a flare [4].

The ‘Dose REduction Strategy of Subcutaneous TNF inhibitors’ (DRESS) study was the first randomized controlled study in RA investigating disease activity–guided dose optimization of adalimumab or etanercept compared with continuation over a period of 18 months [5]. In this study, non-inferiority regarding major flare risk between the groups was shown, and dose reduction or discontinuation was found to be possible in the majority of patients in the dose optimization group. Interestingly, radiographic progression was slightly higher in the dose optimization group and, specifically in this group, was associated with increased disease activity, but not with TNFi dose [5, 6].

The extension of the DRESS study investigated the effectiveness and safety of dose optimization up to 3 years, with dose optimization allowed for both groups [7]. No differences were seen in terms of major flare incidences or disease activity, nor in radiographic progression. Moreover, the cost-effectiveness of dose optimization was confirmed [8]. Following the DRESS study, the effectiveness of TNFi dose optimization has been confirmed in several systematic reviews [9], and supporting data also exist on the tapering of other DMARDs [10, 11] and in other inflammatory diseases [12–14]. Consequently, for RA, a recommendation on dose optimization was included in the current EULAR guideline, stating that in patients in sustained remission without glucocorticoid use, dose reduction of any DMARD [biologic DMARDs (bDMARDs), targeted synthetic DMARDs (tsDMARDs) and/or conventional synthetic DMARDs (csDMARDs)] may be considered [1].

Because of the chronic nature of RA, longer-term data (>3 years) on dose optimization are of importance. The most important outcomes of interest for dose optimization would be disease activity, b/tsDMARD use, radiological outcomes, and the interrelation between these three key variables. Also, it is debated whether a discontinuation attempt should be part of a dose optimization strategy [1], and it is not clear whether repeated dose optimization attempts over time are a sensible approach. Therefore, this study aimed to assess 10-year outcomes of TNFi dose optimization in RA in a cohort of patients originally included in the DRESS study.

## Materials and methods

### Study design and participants

This is a 10-year observational extension study of the DRESS study, a randomized controlled, open-label, non-inferiority trial that compared disease activity–guided dose optimization of TNFi with dose continuation in patients with RA. An extensive description of the inclusion criteria and the rationale of the study are available elsewhere [5, 15]. In short, RA patients treated with stable adalimumab or etanercept for ≥6 months with stable low disease activity (LDA) on at least two consecutive visits were included.

For the current study, participants of the DRESS study were included if they had completed the 3-year extension study. We collected pseudonymized data on patient, disease and treatment characteristics from the electronic health records from the Sint Maartenskliniek (locations Nijmegen, Boxmeer, Geldrop and Woerden) from January 2015 to October 2022, and combined this with data from the earlier publications. The local ethics committee provided exemption for this follow-up study (METC Oost-Nederland; 2023–16202), as ethical approval for this type of study is not required under Dutch law.

### Procedures

During the 18-month intervention phase, patients were treated following a standardized treat-to-target protocol, with visits every 3 months [5, 15]. Disease activity was assessed with the DAS28-CRP [16]. Patients in the dose optimization group with DAS28-CRP ≤ 3.2 received stepwise dose reduction by increasing the interval of adalimumab or etanercept. The dose optimization protocol contained the following steps, displayed as percentage of the current daily dose to the defined daily dose (%DDD): 100%—66%—50%—0% (full discontinuation). In case of a flare, the dose was increased to the last effective dose or, in the case of a flare at full dose, treatment was switched to another biologic DMARD (bDMARD). Flare definition was a DAS28-CRP increase from baseline of >1.2, or as an increase of >0.6 with a current DAS28-CRP of ≥3.2 [16].

During the extension phase (months 18–36), disease activity–guided dose optimization was encouraged for all participants. After the extension phase, it became standard of care for all RA patients in the Sint Maartenskliniek with sustained LDA or remission [17]. The dose optimization protocol was slightly adjusted, with an extra step (33% of the DDD) before discontinuation, resulting in the following protocol: 100%—66%—50%—33%—0%. Also, since March 2015, the more stringent cut-offs validated for DAS28-CRP for remission (DAS28-CRP < 2.4) and LDA (DAS28-CRP < 2.9) have been used [18]. During the observational follow-up, patients were treated by their own rheumatologist, and treatment changes were based on shared decision-making between patient and rheumatologist.

### Outcomes

For this extension study, we defined the following descriptive study outcomes: (1) TNFi dose, b/tsDMARD dose and disease activity over time, (2) proportions of patients with a first and second dose optimization attempt, and the effectiveness of those attempts, (3) proportion of patients with, and duration of the first discontinuation attempt, and (4) radiographic progression between 3 and 10 years.

The TNFi and other b/tsDMARD dose over time was defined as the mean time-weighted ratio of the current dose to the DDD per subsequent year after baseline. Adalimumab 40 mg/2 weeks, and etanercept 50 mg/week were used as 100% of the DDD (Supplementary Table S1, available at *Rheumatology* online). We used the trapezoid method to calculate the mean time-weighted drug use. Drug survival was defined as the use of the current b/tsDMARD until start of a new b/tsDMARD or censoring. Time after discontinuation of the current b/tsDMARD was still considered drug survival, including the start of a glucocorticoid and/or a csDMARD, as long as no other b/tsDMARD was started. The disease activity over time was defined as the mean time-weighted DAS28-CRP, also calculated with the trapezoid method.

For both the dose optimization and discontinuation attempts, only the first episode of the original DRESS TNFi use was used (until switch to another b/tsDMARD). A dose optimization attempt was defined as the moment of initiation of the dose optimization protocol up until the first dose increase or censoring. For example, when a patient was using the full dose of the TNFi (100% of the DDD) at the start of the study, the moment of starting 66% of the DDD was marked as the start of the dose optimization attempt. If the same patient was using 66% of the DDD after the first dose optimization attempt (due to a persistent flare at 33%) but re-attempted 33% of the DDD at a later time point, the initiation of 33% was also marked as a dose optimization attempt. The effectiveness of dose optimization attempts were operationalized as change in the %DDD after 1.5 years (in line with the extension study). Additionally, we calculated the proportions of patients with a lower dose, full discontinuation, and stable dose.

The first discontinuation attempt was defined as reaching a %DDD of 0% for the first time. The duration of a discontinuation attempt was defined as the time from reaching 0% until restart of the same or another b/tsDMARD. Use of concomitant antirheumatic drugs (csDMARDs, NSAIDs, glucocorticoids) was allowed and noted.

We assessed radiographic progression of the hands and feet between 3 years and 10 years, using the radiographs taken at the end of the DRESS extension study as the 3-year time point [7], and radiographs taken (in routine care) between June 2021 and January 2023 as the 10-year time point. The radiographs were scored pairwise by two readers (C.J.T.v.d.T. and N.v.H.), blinded for disease activity and medication use, but in a known sequential order, using the modified Sharp–van der Heijde score (SvdH, range 0–448) [19]. The 3-year radiographs were rescored for this study. We calculated the mean progression in SvdH between 3 and 10 years and per year, as well as the proportion of patients exceeding (1) the smallest detectable change (SDC), calculated with the 95% levels of agreement method [20] and (2) a score of 0.5 SvdH units representing minimal radiographic progression [5]. In addition, as we found a relationship between radiographic progression and mean time-weighted DAS28-CRP with the effect modification of TNFi %DDD in the first 1.5 years of the DRESS study [6], we studied the relationship between disease activity on progression, and b/tsDMARD dose on progression, in a multivariable logistic regression analysis containing all three variables.

### Statistical analysis

We performed no formal sample size analysis, as we used all eligible DRESS study participants. The last available DAS28-CRP measurement of each patient before 1 November 2022 was used as the censoring date. For the exploratory outcomes, descriptive statistics of mean ± S.D. and median (interquartile range) were used, depending on their distribution. For percentages, the 95% CIs were calculated where appropriate.

Durations of drug survival and discontinuation were analysed with Kaplan–Meier analyses. For radiographic progression, logistic regression was used for studying the relationship between radiographic progression, %DDD and disease activity. Progression exceeding the SDC was used as the cut-off point (progression yes/no) for the dependent variable, with mean time-weighted DAS28-CRP and b/tsDMARD %DDD as independent variables. The results were displayed as odds ratios (ORs) with 95% CIs. Stata/IC version 13.1 (StataCorp, College Station, TX, USA) was used for the statistical analyses.

## Results

### Participants

**Figure 1.**
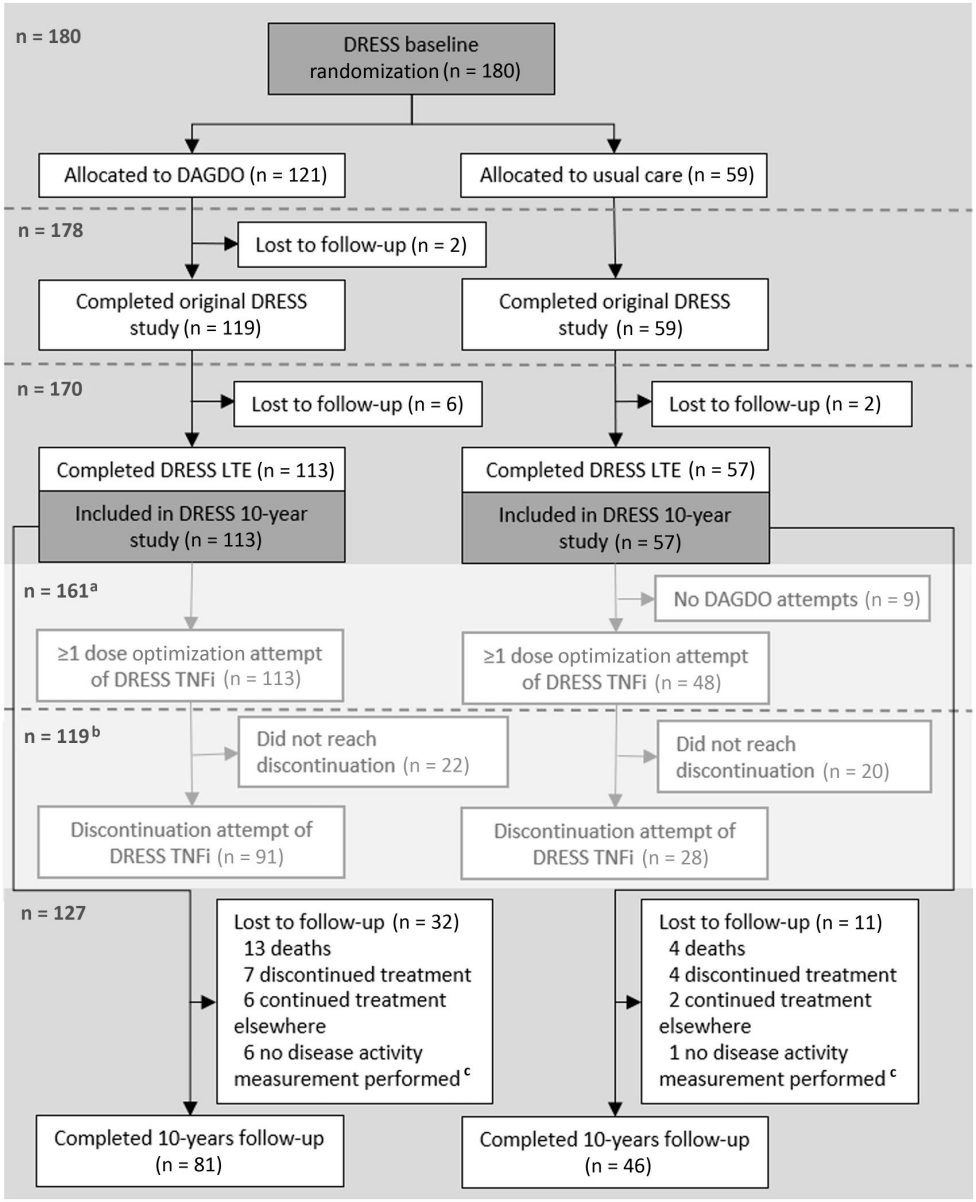
Study flowchart. ^a^Represents the number of patients with ≥1 DAGDO attempt. ^b^Represents the number of patients with ≥1 discontinuation attempt. At least one disease activity measurement was required to be included in the analyses. DAGDO: disease activity–guided dose optimization; DRESS: Dose REduction Strategy of Subcutaneous TNF inhibitors study; LTE: long-term extension study (3 year follow-up); TNFi: TNF inhibitor

Of the 180 patients randomized in the original DRESS-study, 170 patients (94%) completed the 36 months follow-up (original randomization: 113 disease activity–guided dose optimization and 57 usual care). No objections against pseudonymous data use were received; thus, the data of all 170 patients could be included in this study (Fig. 1). A total of 127 patients completed the 10-year follow-up (Fig. 1), and the median follow-up time was 10.0 years [interquartile range (IQR) 9.3–10.3]. The patient characteristics are displayed in Table 1.

**Table 1.** Baseline characteristics

	Total (*n* = 170)
**General characteristics**	
Female sex	109 (64)
Age at baseline (years)	59 ± 10
Active smoker at baseline	44 (26)
BMI at baseline (kg/m^2^)	26.7 ± 4.6
**Disease characteristics**	
Disease duration at baseline (years)	10 (6–16)
RF positive	136 (80)
ACPA positive	124 (73)
Erosive disease at baseline	96 (61, *n* = 158)
DAS28-CRP score at baseline	2.18 ± 0.69
**Treatment characteristics**	
TNFi at baseline (etanercept/adalimumab)	112/58
Duration of DRESS TNFi use (years)	3.4 ± 2.4
Concomitant csDMARD use at baseline	113 (66)
≥1 previous TNFi used	62 (37, *n* = 166)
DRESS randomization (dose optimization/usual care)	113/57

Either displayed as mean ± S.D., median (interquartile range) or number (percentage). TNFi: TNF inhibitor; DRESS: Dose REduction Strategy of Subcutaneous TNF inhibitors study.

### Medication use and disease activity

At DRESS-study baseline, the used TNFi (DRESS TNFi) was etanercept for 112 patients and adalimumab for 58 patients. The median drug survival of the DRESS TNFi from study baseline was 8.9 years (min to max: 0.50–10.7 years) and was similar for etanercept (median 8.5 years) and adalimumab (median 9.3 years). At their last available measurement, 55 patients (32%) were still using their DRESS TNFi, 60 patients (36%) used another b/tsDMARD and another 55 patients (32%) had discontinued their DRESS TNFi without starting a new b/tsDMARD. Of the 55 patients without a b/tsDMARD at that time, 29 patients were using a csDMARD, 2 patients oral prednisolone, 4 patients both a csDMARD and prednisolone, and 20 patients were DMARD-free. Throughout the study, 60 of 170 patients changed to a different b/tsDMARD from their original DRESS TNFi.

**Figure 2.**
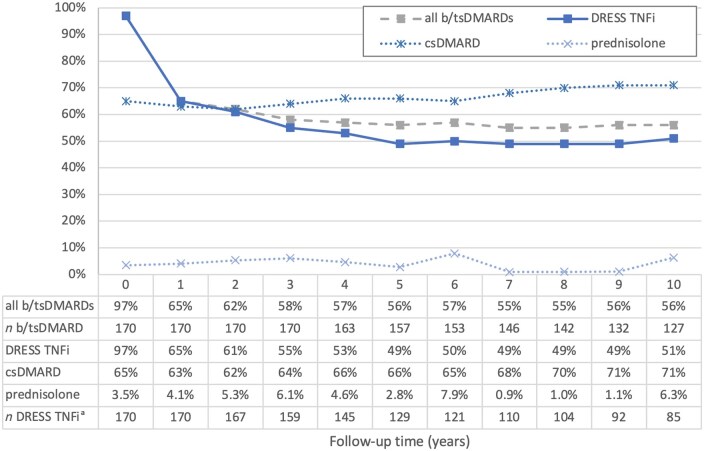
Represents the drug dose relative to the daily defined dose (%DDD) per subsequent year (for all b/tsDMARDs and DRESS TNFis), and the proportion of patients on DRESS TNFi using co-medication (for csDMARD and prednisolone). ^a^Number also applicable for csDMARD and prednisolone. b/ts DMARD: biologic/targeted synthetic DMARD; csDMARD: conventional synthetic DMARD; DRESS: Dose REduction Strategy of Subcutaneous TNF inhibitors study; TNFi: TNF inhibitor

The proportion of the mean DRESS TNFi dose in relation to the %DDD decreased from 97% at baseline (95% CI 96–99%, *n* = 170)% to 49% at year 5 (95% CI 42–56%, *n* = 129, see Fig. 2). At year 10, the %DDD remained stable: 51% (43–59%, *n* = 85). The same pattern was found for all b/tsDMARDs (including the DRESS TNFi), with a decrease from 97% at baseline (96–99%, *n* = 170) to56% at year 10 (49–63%, *n* = 127, see also Fig. 2).

**Figure 3.**
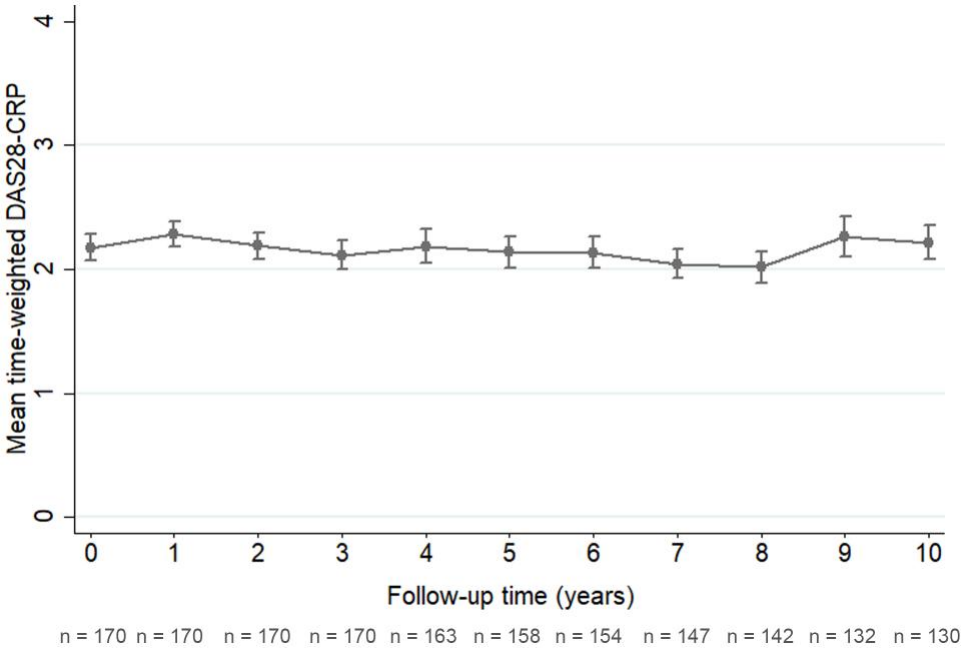
Mean time-weighted disease activity per year measured with DAS28-CRP

The mean time-weighted disease activity measured with DAS28-CRP throughout the study was 2.13 (95% CI 2.10–2.16). An overview of the mean time-weighted DAS28-CRP per study year is displayed in Fig. 3.

### Dose optimization attempts

One hundred and sixty-one patients (161/170, 95%) had at least 1 dose optimization attempt of their DRESS TNFi during the study. The median number of dose optimization attempts per patient was 2 (range 0–5). Of the 161 patients, 159 had at least 1.5 years of follow-up of their first dose optimization attempt available. When comparing the DRESS TNFi dose after 1.5 years with the dose at the start of the first optimization attempt in these patients, 70 used a lower dose (44%), 46 had reached full discontinuation (29%), and 43 used a similar dose (27%). The mean %DDD reduction was 48% ± 38%.

Ninety-nine patients (99/161, 61%) had a second dose optimization attempt. The median time between the start of the first and second attempt was 2.7 years (IQR 1.7–4.2 years). The starting dose for the second dose optimization attempt was full dose (%DDD = 100%) for 60 patients (61%) and a tapered dose (%DDD < 100%) for 39 (39%). The time between the first and second attempt was not associated with a successful second attempt (no restart or dose increase during study period; OR 0.92, 95% CI 0.73–1.18). Of the 99 patients, 89 had at least 1.5 years of follow-up of their second dose optimization attempt available. When comparing the DRESS TNFi dose after 1.5 years with the dose at the start of the second optimization attempt in these patients, 44 used a lower dose (51%), 18 had reached full discontinuation (21%), and 24 used a similar dose (28%). The mean %DDD reduction was 33% ± 37%.

### Discontinuation attempts

Of the 161 patients with a dose optimization attempt, 119 (74%) attempted discontinuation of the DRESS TNFi at least once (Fig. 1). At the time of their first discontinuation attempt, 65 patients (56%, 65/119) used a csDMARD as co-medication, 3 oral glucocorticoids (3%) and 2 both a csDMARD and an oral glucocorticoid (2%). The median glucocorticoid dose at that time was 5 mg daily (*n* = 5), with one patient being on a short course of 30 mg for 7 days.

**Figure 4.**
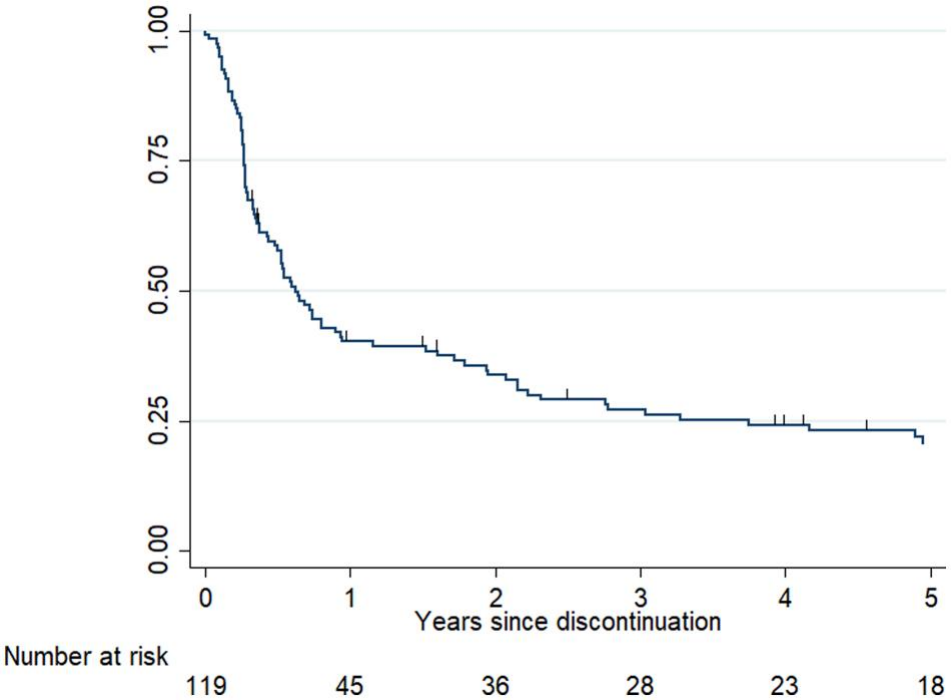
Survival of first discontinuation attempt of the DRESS TNFi in years. The upticks represent censored subjects. DRESS: Dose REduction Strategy of Subcutaneous TNF inhibitors study; TNFi: TNF inhibitor

The median duration of the first discontinuation attempt was 7 months (IQR 3–33 months). The survival of the first discontinuation attempt is displayed in Fig. 4. Thirteen patients (11%, 13/119) started extra co-medication during this discontinuation period: 10 patients started a csDMARD and 3 started oral glucocorticoids.

Twenty-five patients (21%, 25/119) never had to restart their TNFi or another b/tsDMARD after their first discontinuation attempt, throughout the study period. The median observed time in discontinuation for these patients was 73 months (IQR 30–111 months). At the end of follow-up, 12 of these patients (48%) were using a csDMARD, 1 an oral prednisolone (4%), and 12 were DMARD-free (48%).

### Radiographic joint damage

Eighty-six patients had radiographs at both year 3 and 10 available. The median SvdH score at 3 years was 25.8 units (IQR 10.0—53.5). The median progression was 5.5 units (IQR 2.5–13.0, see Table 2). Forty-one patients (48%) had a progression exceeding the SDC (5.7 units), and 78 patients (91%) had a progression exceeding the minimal radiographic progression (0.5 units, Supplementary Fig. S1, available at *Rheumatology* online).

**Table 2.** Radiographic outcomes between 3 and 10 years of follow-up

	Study participants (*n* = 86)
Progression total SvdH score	5.5 (2.5–13)
Progression SvdH score per year	0.8 (0.4–1.8)
Progression erosion score	2 (0.5–5.5)
Progression joint space narrowing	3.5 (1.5–8.5)
Progression > SDC (5.7 units)	41 (48)
Progression > 0.5 units	78 (91)

Either displayed as median (IQR) or number (percentage). SvdH: Sharp–van der Heijde; SDC: smallest detectable change.

In the regression analysis, a higher mean time-weighted DAS28-CRP was significantly associated with reaching progression equal to the SDC or more (OR 3.71 per increased point of DAS28-CRP, 95% CI 1.31–10.56, *P* = 0.014), whereas a relevant association with the b/tsDMARD %DDD could not be demonstrated (OR 0.42 for a %DDD of 100% compared with 0%, 95% CI 0.07–2.49, *P* = 0.34).

## Discussion

This 10-year study on the effectiveness and safety of disease activity–guided dose optimization of TNFis in RA patients showed four key results that are reassuring but require some attention. First, we found a stable LDA over a 10-year period while only using half of the TNFi dose. Second, a subsequent dose reduction attempt also led to a relevant reduction in b/tsDMARD use, albeit less reduction compared with the first attempt. Third, the inclusion of a discontinuation attempt in the dose optimization strategy seems sensible, as it did not lead to long-term disease deterioration, and because b/tsDMARD-free remission for a relevant period is possible in a non-negligible number of patients. Last, there was a relevant progression of joint damage for half of the patients, although limited, which was not associated with bDMARD use or dose, but seemed partly driven by higher disease activity.

Our study has several strengths. It is the largest study on disease activity–guided dose optimization of b/tsDMARDs in RA, with the longest follow-up time and with solid data quality and low attrition. Also, treat-to-target was adequately performed in this study, demonstrated by several indicators of protocol adherence. This also enabled us to analyse the effects of dose and disease activity independently.

However, our study has some limitations. First, especially for the radiographs, there was a significant proportion of data missing (49% missing). This reduced precision and might have induced bias, as radiographs possibly have been performed more often in patients with more complaints and/or more active disease who were more likely to visit the clinic, leading to an overestimation of the radiographic progression. Of note, the proportion of missing data for the clinical outcomes (disease activity and b/tsDMARD dose) was low for this retrospective design and long follow-up duration. Second, some DAS28-CRP measurements were missing at the start of dose optimization or discontinuation. Assuming that the measurements were performed more often in the presence of complaints, the disease activity may therefore be somewhat overestimated. Last, the precision was not always enough to exclude all relevant effect sizes, especially for the radiographic outcomes.

To interpret the radiographic progression seen in our study, there is no suitable direct control group available. Recent studies reporting radiographic progression in RA in cohorts with ≥5 years of follow-up without dose optimization found a somewhat lower mean progression than our study: 1.8–3.1 SvdH units for 5 years follow-up and 2.5–3.8 SvdH units for 10 years follow-up, compared with 5.5 SvdH units in our study [21, 22]. However, this difference seems mainly due to the higher joint space narrowing subscore in our study compared with other studies [22–24], which is probably caused by a much longer disease duration at baseline in our study (10 years *vs* 5–8 months), resulting in greater effect of primary OA on the progression score. Of note, the median progression found of 5.5 SvdH units over 7 years was approximately the same as the previously suggested minimal clinically important difference (MCID) for 1 year progression [25]. Similarly to the original DRESS study, we found an association between radiographic progression and a higher DAS28-CRP, suggesting that radiographic progression is driven by disease activity [6]. Although we found no significant effect of b/tsDMARD dose on radiographic progression in this study, a smaller effect cannot be ruled out because of the limited sample size, and therefore this requires further study.

Although this is a topic of debate, our study found indications of a positive risk–benefit ratio for the inclusion of discontinuation in a dose optimization attempt. This is demonstrated by the stable LDA over time, the long drug survival after restart, and the finding that one-fifth of patients remained without a TNFi for multiple years. The perception that discontinuation is a suboptimal strategy mainly stems from randomized trials in which direct discontinuation from a full dose without the opportunity to restart was inferior to continuation of full dose, as well as trials with short-term flares as the primary outcome [26, 27]. However, a strategy of stopping and restarting when needed is different from stopping without taking the effects of restarting into account, and this difference should be appreciated. Therefore, we recommend a careful stepwise dose reduction with adequate monitoring of disease activity for the selection of a subgroup of patients in whom a discontinuation attempt might be fruitful.

Another interesting finding is the additional value of a subsequent dose optimization attempt after ∼2.5 years. Although the mean dose reduction was lower in the follow-up attempt (33% *vs* 48%), the proportion of patients after 1.5 years on either a lower dose or full discontinuation was similar for both attempts (73% *vs* 72%). The effectiveness of the subsequent attempt suggests that the b/tsDMARD need over time may vary in a patient, possibly caused by a fluctuation in disease severity. Of note, this finding could also be explained by patient and/or physician factors, such as stricter requirements for dose escalation (not in the case of subjective complaints), or more positive beliefs about dose optimization. All in all, we suggest a follow-up dose optimization attempt in all patients after ∼2.5 years, as it has shown to be safe and can lead to additional dose reduction.

While we investigated several aspects of the long-term effectiveness of disease activity–guided dose optimization, some research gaps remain. A possible beneficial effect of long-term dose optimization on adverse effects of TNFi such as infections could be of importance. Also, the long-term effectiveness of disease activity–guided dose optimization for other drugs and diseases would be of interest, as it has been shown to be effective for other drugs in RA [10, 11, 28], as well as in other diseases [10, 12–14].

In conclusion, over a period of 10 years, disease activity–guided dose optimization of TNF inhibitors in RA leads to significant dose reduction while maintaining disease control. A discontinuation attempt seems sensible to include, and subsequent dose optimization attempts after ∼2.5 years can lead to additional dose reduction. A strict treat-to-target approach would appear to be important for limiting radiographic progression. These findings are important for guiding more specific dose optimization recommendations in the future regarding how to perform dose optimization, and how to monitor outcomes to ensure safety.

## Supplementary Material

keae103_Supplementary_Data

## Data Availability

Data are available upon request. Researchers who are interested in doing additional analyses using these data can contact A.A.d.B. via a.denbroeder@maartenskliniek.nl. Data can only be used for scientific research without conflicts of interests.
