# Supplementary material for: Disease activity–guided dose optimization including discontinuation of TNF inhibitors in rheumatoid arthritis is effective for up to 10 years: an observational follow-up of the DRESS study
Source: Rheumatology (Oxford). 2024 Feb 12;64(2):533–40. doi: 10.1093/rheumatology/keae103 (PMC11781578; doi:10.1093/rheumatology/keae103)
Supplement: keae103_Supplementary_Data [file keae103_supplementary_data.docx]

# Supplementary data DRESS 10 year study

**Supplementary Table S1: daily defined dose of the included biological and targeted synthetic DMARDs**

| Biological DMARDs | |
| --- | --- |
| Abatacept | 125 mg every 7 days |
| Adalimumab | 40 mg every 14 days |
| Certolizumab pegol | 200 mg every 14 days |
| Etanercept | 50 mg every 7 days |
| Golimumab | 50 mg every 30 days |
| Rituximab | 1000 mg every 182 days |
| Sarilumab | 200 mg every 14 days |
| Tocilizumab | 162 mg every 7 days |
| Targeted synthetic DMARDs | |
| Baricitinib | 4 mg every day |
| Tofacitinib | 10 mg (2*5) every day |

**Supplementary Figure S1: probability plot**


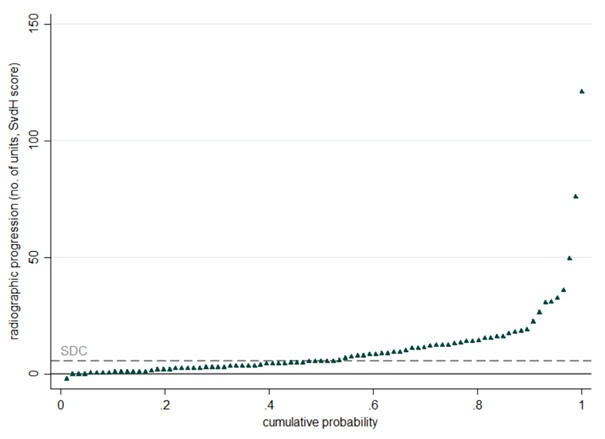


*SDC: smallest detectable change, SvdH: Sharp-van der Heijde. Solid line: no progression, broken line: SDC of 5.7 points, triangle line: probability.*
